# Prolonged avoidance training exacerbates OCD-like behaviors in a rodent model

**DOI:** 10.1038/s41398-020-00892-5

**Published:** 2020-07-03

**Authors:** Freddyson J. Martínez-Rivera, Marcos J. Sánchez-Navarro, Carlos I. Huertas-Pérez, Benjamin D. Greenberg, Steven A. Rasmussen, Gregory J. Quirk

**Affiliations:** 1 Departments of Psychiatry and Anatomy & Neurobiology, School of Medicine, Medical Sciences Campus, University of Puerto Rico, San Juan, PR 00936 USA; 2 Department of Psychiatry and Human Behavior, Alpert Medical School of Brown University and Butler Hospital and the Providence VA Medical Center, Providence, RI 02906 USA; 3 Present Address: Nash family Department of Neuroscience and Friedman Brain Institute, Icahn School of Medicine at Mount Sinai, New York, NY 10029 USA

**Keywords:** Learning and memory, Psychiatric disorders, Learning and memory, Psychiatric disorders, Learning and memory

## Abstract

Obsessive-compulsive disorder (OCD) is characterized by compulsive behaviors that often resemble avoidance of perceived danger. OCD can be treated with exposure-with-response prevention (ERP) therapy in which patients are exposed to triggers but are encouraged to refrain from compulsions, to extinguish compulsive responses. The compulsions of OCD are strengthened by many repeated exposures to triggers, but little is known about the effects of extended repetition of avoidance behaviors on extinction. Here we assessed the extent to which overtraining of active avoidance affects subsequent extinction-with-response prevention (Ext-RP) as a rodent model of ERP, in which rats are extinguished to triggers, while the avoidance option is prevented. Male rats conditioned for 8d or 20d produced similar avoidance behavior to a tone paired with a shock, however, the 20d group showed a severe impairment of extinction during Ext-RP, as well as heightened anxiety. Furthermore, the majority of overtrained (20d) rats (75%) exhibited persistent avoidance following Ext-RP. In the 8d group, only a minority of rats (37%) exhibited persistent avoidance, and this was associated with elevated activity (c-Fos) in the prelimbic cortex and nucleus accumbens. In the 20d group, the minority of non-persistent rats (25%) showed elevated activity in the insular-orbital cortex and paraventricular thalamus. Lastly, extending the duration of Ext-RP prevented the deleterious effects of overtraining on extinction and avoidance. These rodent findings suggest that repeated expression of compulsion-like behaviors biases individuals toward persistent avoidance and alters avoidance circuits, thereby reducing the effectiveness of current extinction-based therapies.

## Introduction

Obsessive-compulsive disorder (OCD) is a chronic illness characterized by compulsive urges resembling avoidance of perceived danger^1^. Behaviorally, OCD is treated with exposure-with-response prevention (ERP) therapy, in which patients are coached to refrain from carrying out compulsions in response to environmental triggers, with the intention of extinguishing those behaviors. Due to their repetitive nature, OCD compulsions have been hypothesized to originate from prolonged and repetitive exposure to avoidance-associated triggers^2,3^. However, little is known about the effects of long-term, repetitive avoidance behavior on subsequent extinction. OCD patients show deficits in fear extinction^4,5^ and goal-directed behaviors^6^, as well as an altered cortico-striatal system^4,6,7^. Extinction impairments are also observed in patients suffering from other psychiatric disorders such as post-traumatic stress disorders (PTSD)^8^, but are not typically accompanied by obsessions^9^. Although ERP is effective^10,11^, compulsions persist for many patients despite treatment^12,13^. Therefore, there is a pressing need to understand how OCD-related circuits and decision-making processes may be altered by long-term repetitive compulsions.

We previously developed a rodent model of ERP therapy^14^ by modifying an active avoidance task in which rats pressing a bar for food can avoid a tone-signaled shock by stepping onto a nearby platform^15^. After conditioning, rats are given several days of extinction training with a barrier inserted to prevent access to the platform (extinction-with-response prevention, Ext-RP). However, the duration of avoidance training in our previous experiment (10 days) may not have been sufficient to have an impact on the development of OCD-like behavior in rats. Extensive conditioning of avoidance may resemble prolonged expression of compulsions, which are thought to be reinforced by the reduction in patients’ stress and anxiety^16^. Therefore, to better approximate the long-term expression of compulsions, we tested the effect of avoidance overtraining (20 days vs. 8 days) on Ext-RP. Following Ext-RP, the activity profile of these two groups were compared using c-Fos indexing to characterize the effects of avoidance overtraining on brain circuits signaling subsequent expression of avoidance.

## Materials and methods

### Subjects and bar-press training

A total of seventy-eight adult male Sprague–Dawley rats (300–380 g; Envigo Laboratories, Indianapolis, IN, USA) were used in this study. Rats were restricted to 18 g/day of standard laboratory chow and trained to press a bar for sucrose pellets on a variable interval schedule of reinforcement averaging 30 s (VI–30 s). Rats were food trained until they reached a criterion of ≥10 presses/min. All procedures were approved by the Institutional Animal Care and Use Committee of the University of Puerto Rico School of Medicine, and the Association for Assessment and Accreditation of Laboratory Animal Care (AAALAC).

### Platform-mediated avoidance and extinction-with-response prevention

Platform-mediated avoidance (PMA) training was performed as previously described^15,17^. Briefly, rats were conditioned with a pure tone (30 s, 4 kHz, 75 dB) co-terminating with an electric shock delivered through a grid floor (2 s, 0.4 mA). Rats received nine tone-shock pairings each day for 8 or 20 days, with a variable inter–trial interval averaging 3 min. An opaque acrylic square platform (14.0 cm each side, 0.33 cm tall) was placed in the opposite corner of the sucrose–delivering bar, to allow rats to avoid the shocks. The platform was present throughout bar-press training to reduce novelty. On either day 9 or day 21, rats received extinction-with-response prevention (Ext-RP) training, in which 15 tones without shocks were delivered per day (Pavlovian extinction), over 4 or 10 days, while access to the platform was blocked with a Plexiglas barrier (Test 1) as previously described (Rodriguez-Romaguera et al.^14^). Following Ext-RP training, the barrier was then removed to assess transfer of extinction learning to avoidance behavior. Behavioral data (position (center of mass), freezing, pressing) was acquired using the Any-Maze tracking system (Stoelting Co).

### Persistence tests

In the first post-Ext-RP test (Test 1), rats were given a single tone with the barrier removed and the time spent on the platform was used as index of persistent avoidance. One hour later, persistent animals were divided into two groups and given a single tone either without the barrier present (Test 2a) or with the barrier placed in the adjacent corner where it did not block entrance to the platform (Test 2b). The following day, persistent rats underwent an additional one-tone test in which both the barrier and the platform were removed (Test 3). Because animals exposed to either the presence or absence of the barrier at Test 2 showed no statistical differences at Test 3 (8d: barrier vs. no barrier: *p* = 0.96; 20d: barrier vs. no barrier: *p* = 0.45), they were combined in their respective main groups and compared (8d vs. 20d).

### Elevated plus maze

To determine whether overtraining induced generalized anxiety, a subset of animals from 8d and 20d groups were tested in an elevated plus maze (EPM) 60 min following Test 1^18^. Subjects were placed in the center of the EPM facing an open arm, while the time spent in open vs. closed arms was recorded for 5 min using the Any-Maze tracking system (Stoelting Co).

### Immunohistochemistry

c-Fos Immunohistochemistry (IHC) was performed as previously described^15,19^ with some modifications. One hour after Test 1, a subset of rats was anesthetized with sodium pentobarbital (450 mg/Kg, i.p.) and transcardially perfused with 250 ml of 0.9% saline followed by 500 ml of cold fresh 4% paraformaldehyde (PFA) in 0.1 M potassium phosphate buffer (PBS) at pH 7.4. Brains were removed and transferred to 30% sucrose in 0.1 M PBS for 48–72 h, for cryoprotection. Using a cryostat (CM 1850; Leica), frozen sections (40 µm) were prepared at the levels of prefrontal/orbital cortices, striatum, and amygdala/thalamus. Sections were initially washed (PBS) and blocked in a solution of 2% normal goat serum (NGS, Vector Laboratories) and 0.1% tween (Tween-20, Sigma-Aldrich) in 0.1 M PBS (pH 7.4) for 1 h. The sections were then incubated overnight at room temperature (RT) with rabbit anti-c-Fos (Ab-5, Oncogene Science) at a concentration of 1:20,000. Sections were washed and then incubated for 2 h at RT in a solution of biotinylated goat anti-rabbit IgG (1:200; Vector Laboratories) and placed in a mixed avidin-biotin horseradish peroxidase complex solution (ABC Elite Kit, Vector Laboratories) for 90 min. Black immunoreactive nuclei labeled for c-fos were visualized after 5 min of exposure to DAB/peroxidase substrate kit (Vector Laboratories). Sections were mounted on coated-gelatin slides, dehydrated and cover slipped. Counter sections were stained with Nissl and used to determine the anatomical boundaries of each structure analyzed.

c-Fos labeled neurons were automatically counted at 20x magnification with an Olympus microscope (Model BX51) equipped with a digital camera. We restricted our analysis to several areas previously implicated in conditioned fear or avoidance behavior^14,15,17,20^. Images were generated for subregions of prefrontal cortex (+3.72 to 2.52 AP), orbitofrontal cortex (+4.20 to +3.00 AP), anterior agranular insular cortex (+4.20 to +3.00 AP), dorsal striatum (+2.16 to +1.20 AP), nucleus accumbens (+2.04 to +1.20 AP), basal nucleus of the amygdala (−2.28 to −3.12 AP), central nucleus of the amygdala (−2.28 to −2.92 AP), paraventricular thalamus (−2.28 to −3.12 AP)^21^. Positive c-fos-like immunoreactivity showed brown-black staining distinct from the background. c-fos positive cells were automatically counted and averaged (blinded) for each hemisphere at 2–4 different rostro-caudal levels of each structure using Metamorph software 6.1 (Molecular Devices). The density of Fos labeling was calculated by dividing the number of positively labeled neurons by the total area of each region (counts/0.1mm^2^).

### Data analysis

Student *t*-tests (two-sided), Fisher exact tests, and ANOVAs were performed to determine statistical differences in behavioral and immunohistochemistry experiments. For post hoc tests, we used Tukey (behavior) or Fisher LSD (c-Fos). Pearson correlation analyses were also performed. Data is presented as mean ± standard error (SEM) and statistical significance was established as *p < 0.05 (a minimum of subjects were used to achieve significance). All statistical analyses were performed using Statistica 6.0 (Statsoft®) and Prism 6.0 (GraphPad Inc.) software’s.

## Results

### Avoidance behavior was similar following 8d or 20d of conditioning

**Fig. 1:**
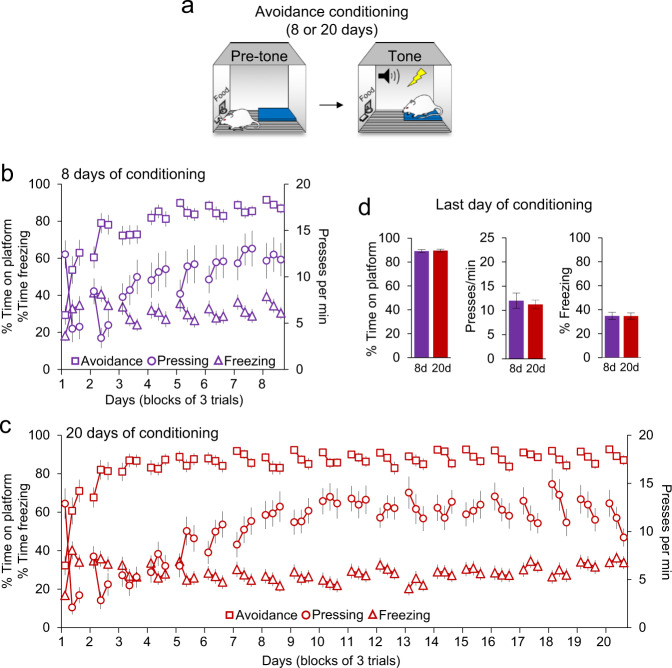
Avoidance conditioning lasting 8d vs. 20d. **a** Rats pressing for sucrose pellets were trained to avoid a tone signaled foot-shock by stepping onto a nearby platform. **b** One group (8d) was conditioned over 8 days. As avoidance increased, freezing to the tone gradually decreased and bar pressing during the pre-tone interval gradually returned to preconditioning levels. **c** A different group (20d) was conditioned over 20 days. **d** 8d and 20d groups showed equivalent levels of avoidance, pressing, and freezing at the end of conditioning. 8d group: *n* = 38; 20d group: *n* = 40. All data are shown of blocks of three trials as mean ± SEM.

Food-deprived rats were separated (randomly) into two groups to receive either standard avoidance conditioning over 8 days (8d group) or extended avoidance conditioning over 20 days (20d group) (Fig. 1a–d). Rats learned to avoid a tone-signaled footshock by stepping onto a nearby platform^15^. Because the platform was positioned away from the food bar, avoidance incurred a cost of lost access to food. On the final day of conditioning, both 8d and 20d groups exhibited similar levels of avoidance expression, as indicated by the percent of the 30 s tone spent on the platform (8d: 89.1%, 20d: 89.6%), as well as the rate of bar pressing during the pre-tone interval (8d: 12 presses/min, 20d: 11 presses/min), and the amount of freezing to the tone (8d: 34.7%, 20d: 34.6%). Thus, following conditioning, 8d and 20d groups were behaviorally indistinguishable in this task.

### The 20d group showed impaired extinction and persistent avoidance

**Fig. 2:**
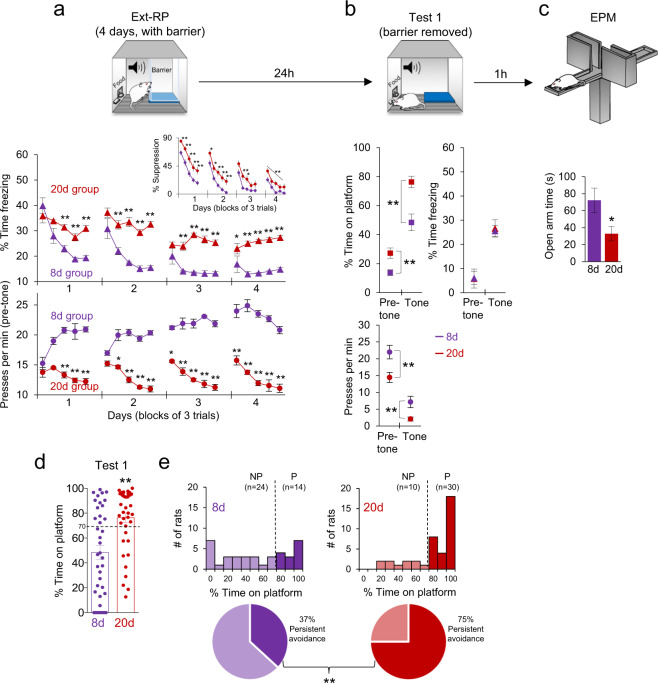
Overtraining leads to impaired extinction (Ext-RP) and persistent avoidance. **a** Rats conditioned for 8d or 20d were presented with tones in the absence of shock over 4 days, with a Plexiglas barrier inserted to block access to the platform (Ext-RP). Initially, both groups showed equivalent freezing, but extinction of freezing was impaired in the 20d group, as was extinction of suppression of bar pressing (inset). Rats in the 20d group were also impaired in their extinction of press suppression during the pre-tone period. **b** One day following Ext-RP training, rats were tested with the barrier removed (Test 1). The 20d group showed excessive avoidance compared to the 8d group, during both the pre-tone and tone periods, but there was no group difference in freezing. **c** The 20d group also showed elevated anxiety, as indicated by reduced time spent in the open arms of an elevated plus maze (EPM). **d**, **e** Distribution of avoidance values (time on platform) during Test 1. Rats showing more than 70% time on platform were classified as persistent (P), whereas those showing less than 70% were classified as non-persistence (NP). 8d group: *n* = 38; 20d group: *n* = 40. For EPM, 8d group: *n* = 20; 20d group: *n* = 14. Ext-RP data shown as blocks of three trials, whereas Test 1 data shown as single trial. All data are mean ± SEM. *p < 0.05, **p < 0.01.

Following avoidance conditioning, rats received 4 days of extinction-with-response prevention (Ext-RP) training, in which tones were delivered without shocks in the presence of a Plexiglas barrier blocking access to the platform^14^. During Ext-RP, repeated measures (RM) ANOVAs followed by Tukey post hoc analyses revealed that the 20d group was severely impaired in extinction of conditioned freezing as compared with the 8d group (*F*_(1, 76)_ = 9.10; *p* < 0.01) (Fig. 2a). On day 4, 20d rats concluded Ext-RP training with significantly elevated levels of freezing compared to the 8d group (*F*_(1, 76)_ = 11.18; *p* < 0.01). The 20d group also showed elevated suppression of bar pressing (F_(1, 76)_ = 13.55; *p* < 0.01) (Fig. 2a; inset) and a reduced rate of spontaneous pressing during the pre-tone interval (F_(1, 76)_ = 20.33; *p* < 0.01) (Fig. 2a; lower panel). Impaired extinction in 20d rats suggests that the lack of access to the platform triggered a generalized fear/anxiety state that persisted throughout Ext-RP.

After Ext-RP, when the barrier was removed (Test 1), the 20d group spent significantly more time on the platform both prior to the tone (*t*_(76)_= 3.23, *p* < 0.01) and during the tone (*t*_(76)_ = 4.03, *p* < 0.01) compared to the 8d group (Fig. 2b). However, avoidance at Test 1 was significantly reduced compared to the last day of conditioning for both groups (both *p*’s < 0.01; *t*-tests). Similarly, the 20d group showed a decreased rate of lever pressing both prior to the tone (*t*_(76)_ = 3.00, *p* < 0.01) and during the tone (*t*_(76)_ = 2.86, *p* < 0.01) (Fig. 2b lower panel). While the freezing levels at Test 1 were equivalent for both groups (8d: 26%, 20d: 25%), freezing in the 8d group was increased relative to the end of Ext-RP, likely due to a contextual renewal effect caused by removal of the barrier. A RM ANOVA confirmed these results within and between groups across these time points (pre-tone vs. tone) for all behavioral measures (avoidance, freezing, and presses; Tukey post hoc analyses, all *p*’s < 0.05 with one exception: *p* = 0.06). Following Test 1, a subset of rats were tested in the elevated plus maze (EPM), and the 20d group exhibited elevated anxiety as indicated by reduced time spent in the open arms (*t*_(32)_ = 2.12, *p* = 0.04) (Fig. 2c).

Together, these results indicate that overtraining of active avoidance impairs subsequent extinction, predisposing rats to persistent avoidance. These effects are unlikely due to the 20d group receiving more shocks than the 8d group during conditioning, because the total number of shocks received (due to failed avoidance trials) did not significantly differ between groups (8d: 18.0; 20d: 20.7; *t*_(76)_= 1.65, *p* = 0.11). Because 8d and 20d groups showed equivalent avoidance, freezing, and pressing at the end of conditioned, this suggests that heightened anxiety in 20d rats was induced by the loss of the avoidance option in the Ext-RP phase. A frequency distribution analysis of avoidance at Test 1 revealed a large subgroup of 20d rats exhibiting ≥70% time on platform (Fig. 2d, e). We therefore used this cut-off value to define persistent avoidance behavior. Accordingly, 37% (*n* = 14) of rats in the 8d group were persistent (P) whereas 63% (*n* = 24) were non-persistent (NP). However, in the 20d group, 75% (*n* = 30) of rats were persistent, whereas 25% (*n* = 10) were not. The ratio of persistence was significantly higher in the 20d group (Fisher’s exact test: *p* < 0.01) (Fig. 2e; lower panels).

### Persistent avoidance in the 20d group could be reduced with a contextual cue

**Fig. 3:**
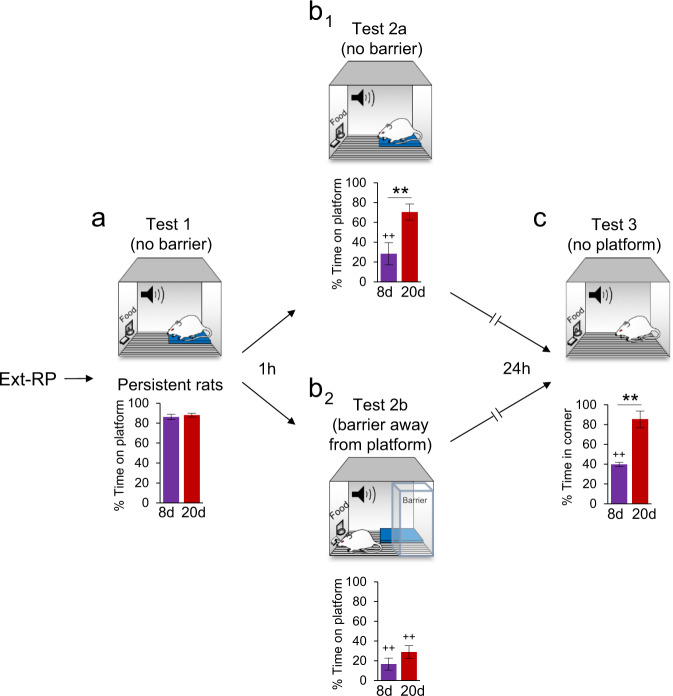
Additional tests for avoidance persistence. **a** Rats classified as persistent avoiders in Test 1 were given additional tests for avoidance. **b**_1_ Simply re-testing the rats one hour later with the barrier still absent reduced avoidance in the 8d group but not in the 20d group. **b**_2_ Re-testing a different subset of rats one hour later with the barrier placed in the adjacent corner (without blocking the platform) reduced avoidance in the 20d group, suggesting that the barrier acted as a contextual occasion setter signaling safety. **c** With both the barrier and platform removed, rats in the 20d (but not the 8d) group spent excessive time where the platform had been located, suggesting persistent avoidance. Test 1, 8d: *n* = 12; 20d: *n* = 31. Test 2 (no barrier), 8d: *n* = 5; 20d: *n* = 14; Barrier (away from the platform), 8d: *n* = 7; 20d: *n* = 17. Test 3 (no platform), 8d: *n* = 8; 20d: *n* = 5. All data are shown as mean ± SEM. **p < 0.01 between group differences; ^++^*p* < 0.01 within group differences as compared with their Test 1.

Subsets of rats from both groups that exhibited persistent avoidance were given two additional tests to assess the durability of their persistent avoidance (Fig. 3a, b). First, they were re-tested one hour after Test 1 with either no barrier present or with a barrier placed away from the platform (adjacent corner), so as not to block access to the platform. We reasoned that the presence of the barrier might act as a contextual occasion setter^22,23^ predictive of the absence of the shock because it first appeared when shocks were omitted.

Accordingly, we performed a two-way ANOVA followed by Tukey post hoc analysis to capture potential differences of 8d and 20d groups through Test 1, Test 2a (no barrier), and Test 2b (barrier). This analysis revealed a significant main effect of Test (*F*_(3,78)_ = 45.15, *p* < 0.01), treatment group (*F*_(1,78)_ = 8.54, *p* < 0.01) and interaction (*F*_(3,38)_ = 3.55, *p* = 0.01), confirming that 8d rats showed a reduction in avoidance from Test 1 to Test 2a (89–28%, *p* < 0.01), whereas no significant reduction was observed in the 20d group (87–70%, *p* = 0.37) (Fig. 3b_1_). Placing the barrier away from the platform significantly reduced avoidance from Test 1 to Test 2b in both groups (8d: 85–17%, *p* < 0.01; 20d: 89–29%, *p* < 0.01) (Fig. 3b_2_), suggesting that persistent avoidance in overtrained rats is sensitive to contextual occasion setters that signal safety^23–25^.

In a final test, we removed both the barrier and the platform, and observed that rats in the 20d group spent increased time in the corner where the platform had been located (RM ANOVA and Tukey post hoc) (Fig. 3c). Comparing Tests 1 and 3 revealed significant effects of Test (*F*_(1,11)_ = 8.20, *p* = 0.01), group (*F*_(1,11)_ = 5.05, *p* = 0.04), and interaction (F_(1,11)_= 6.59, *p* = 0.02). Whereas the 8d group showed a significant reduction in Test 3 (86–39%, *p* < 0.01), the 20d group did not (88–85%, *p* = 0.99). The maintenance of avoidance in the 20d group in the absence of the platform suggests that contextual cues (cage corner) were sufficient to drive avoidance. Thus, persistent avoidance in overtrained animals is long-lasting and is modulated by contextual cues. Notably, no group differences in freezing levels were detected across the tests (ANOVA’s and Tukey post hoc analyses, all *p*’s > 0.05).

### c-Fos revealed structures in the 8d group signaling persistence, and structures in the 20d group signaling non-persistence

**Fig. 4:**
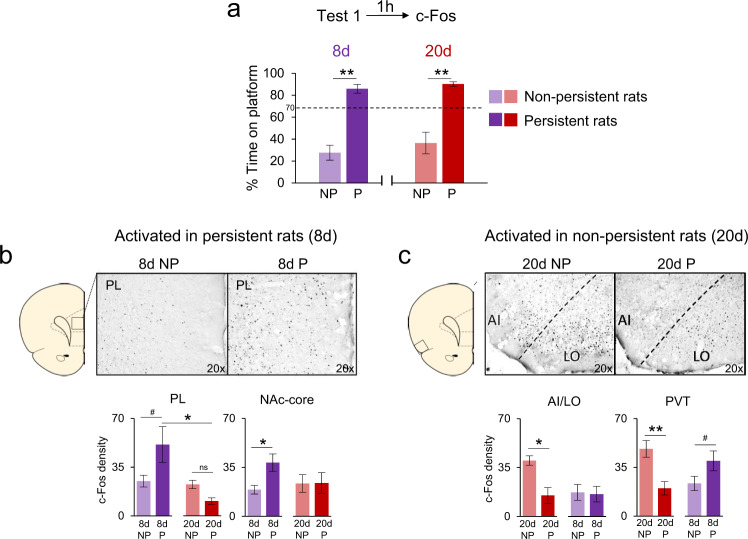
c-Fos signaling of persistent vs. non-persistent avoidance. **a** Avoidance behavior at Test 1 in a subset of animals showing persistent (P) and non-persistent (NP) avoidance. One hour after Test 1, a subset of rats was processed for c-Fos immunocytochemistry. **b** Micrograph shows representative c-Fos labeling in prelimbic (PL) cortex for NP and P rats. 8d group data for c-Fos labeling in PL and nucleus accumbens-core (NAc-core). The 8d group showed elevated c-Fos in P rats compared to NP rats (or to 20d P), suggesting signaling of persistence. **c** In contrast to 8d rats, no structure in the 20d group signaled persistence, but several structures showed elevated activity in non-persistent rats. Micrograph shows representative c-Fos labeling in anterior insular (AI; ventral portion) and lateral orbital (LO; lateral portion) cortices for NP and P rats. Structures signaling non-persistence included AI/LO and paraventricular thalamus (PVT). No structure in the 8d group showed signaling of non-persistence. 8d NP: *n* = 8; 8d *P*: *n* = 4; 20d NP: *n* = 3 (AI/LO)-4; 20d *P*: *n* = 5. All data are shown as mean ± SEM. *p < 0.05, **p < 0.01, #*p* ≤ 0.1, ns non-significant.

To examine differences in neuronal activity between 8d and 20d groups, we obtained c-Fos expression profiles in a subset of rats sacrificed 60 min after Test 1. We initially performed a two-way ANOVA (groups vs. brain regions) comparing c-Fos expression between 8d and 20d groups, however, no group differences were detected (main effect of group: *F*_(1,374)_ = 0.93, *p* = 0.33). We then proceeded to distinguish persistent (P) from non-persistent (NP) subgroups. Table 1 shows c-Fos values for all regions analyzed. A two-way ANOVA (groups × regions) detected a main effect of subgroup (NP vs P; *F*_(3,334)_= 5.32, *p* < 0.01) and brain region (F_(19,334)_ = 16.36, *p* < 0.01), but not an interaction (*F*_(57,334)_ = 0.94, *p* = 0.59), which indicates the substantial variability of c-fos levels among the 4 subgroups and 20 brain regions. Post hoc tests were performed to detect significant differences between subgroups in each region. In the 8d group, the rats showing persistent avoidance exhibited increased activity in areas previously associated with avoidance in this task^15,17^ as compared to rats not showing persistence (one-way ANOVA, *F*_(3,17)_ = 24.67, *p* < 0.01, Tukey post hoc; Fig. 4a). These areas include the nucleus accumbens core (NAc-core, *p* = 0.03) and a trend toward significance in prelimbic cortex (PL, *p* = 0.14) (Fig. 4b). Expressed as a ratio of P/NP groups, PL showed significantly more c-Fos expression at 8d compared to 20d (8d: 153%; 20d: 62%; *t*_(7)_ = 2.55, *p* = 0.03). Surprisingly, however, the persistent rats in the 20d group did not show increased activity in these areas (Fig. 4b). In fact, PL activity in 20d persistent rats was significantly lower than 8d persistent rats (*p* = 0.04; Fig. 4b). Indeed, none of the structures analyzed in the 20d group showed increased activity in the persistent subgroup compared to the non-persistent subgroup (Table 1), suggesting that the persistent avoidance in the 20d group was not mediated by the avoidance circuit previously described for this task.

**Table 1:** Brain structures signaling persistent and non-persistent avoidance behavior.

Region	8d NP	8d P	20d NP	20d P
Prelimbic cortex (PL)	12.5 ± 2.1	25.6 ± 6.4^#+^	11.3 ± 1.5	5.3 ± 1.1
Infralimbic cortex (IL)	17.7 ± 2.2	27.6 ± 6.2	11.7 ± 1.2	9.0 ± 2.1
Medial orbital cortex (MO)	25.4 ± 7.3	17.5 ± 2.4	37.0 ± 14.0^#^	22.6 ± 8.8
Ventral orbital cortex (VO)	18.1 ± 4.4	24.1 ± 8.8	25.7 ± 8.2	15.4 ± 5.6
Lateral orbital cortex (LO), medial part	54.5 ± 13.5	71.8 ± 22.2^#^	67.8 ± 13.3	68.3 ± 26.9
Lateral orbital cortex (LO), lateral part	19.9 ± 6.6	19.6 ± 5.6	41.8 ± 6.1*^+^	16.6 ± 6.3
Agranular insular cortex (AI), rostro-ventral part	14.6 ± 5.7	12.0 ± 4.6	39.5 ± 15.0*^+^	13.4 ± 5.8
Agranular insular cortex (AI), rostro-dorsal part	20.1 ± 5.1^#^	5.8 ± 2.2	6.8 ± 4.2	2.5 ± 1.6
AI/LO area	17.3 ± 5.6	16.0 ± 5.5	40.0 ± 3.3*^+^	15.0 ± 5.6
Nucleus accumbens core (NAc-core)	19.0 ± 3.0	38.3 ± 6.2*	23.4 ± 6.2	23.8 ± 7.3
Nucleus accumbens medial shell (NAc-mshell)	8.4 ± 1.4	21.8 ± 4.6^#^	13.7 ± 4.5	12.7 ± 3.8
Nucleus accumbens lateral shell (NAc-lshell)	12.8 ± 6.2	11.6 ± 6.1	27.0 ± 8.4	21.4 ± 11.6
Dorsomedial striatum (DMS)	2.2 ± 1.6	9.6 ± 3.6	9.2 ± 3.3	2.0 ± 0.7
Dorsolateral striatum (DLS)	0.9 ± 0.4	0.9 ± 0.5	5.3 ± 1.0	2.2 ± 0.6
Basal amygdala (BA)	17.3 ± 2.8	15.1 ± 2.2	14.4 ± 2.8	25.4 ± 11.4
Lateral amygdala (LA)	10.5 ± 1.4	8.7 ± 0.4	10.2 ± 0.6	11.6 ± 2.7
Central amygdala, medial part (CeM)	2.1 ± 0.5	6.1 ± 2.1	5.3 ± 1.4	4.2 ± 2.0
Central amygdala, lateral part (CeL)	5.0 ± 1.4	7.9 ± 1.6	7.3 ± 0.7	6.1 ± 2.4
Mediodorsal thalamus (MD)	4.9 ± 1.4	6.4 ± 0.9	8.4 ± 2.9	4.0 ± 3.5
Paraventricular thalamus (PVT)	23.1 ± 5.0	39.2 ± 7.0^#^	48.9 ± 5.9*^+^	19.9 ± 4.8

NP vs. P: *p < 0.05; ^#^*p* ≤ 0.1.

NP vs. NP or P vs. P:^+^*p* < 0.05.

In contrast to the 8d group, the 20d group showed elevated brain activity in the minority of rats showing non-persistence (*p* < 0.01; Fig. 4a). These structures were: the rostro-ventral portion of the agranular insular cortex (AI: *p* = 0.01), the border of AI with the lateral portion of the lateral orbital cortex (AI/LO: *p* = 0.02), the lateral portion of the lateral orbital cortex (LO: *p* = 0.02), and the paraventricular thalamus (PVT: *p* < 0.01) (Fig. 4c). Thus, activation of cortico-striatal-thalamic areas was associated with the minority of rats in the 20d group that were able to overcome persistence.

### The deleterious effects of overtraining could be prevented by extending Ext-RP

**Fig. 5:**
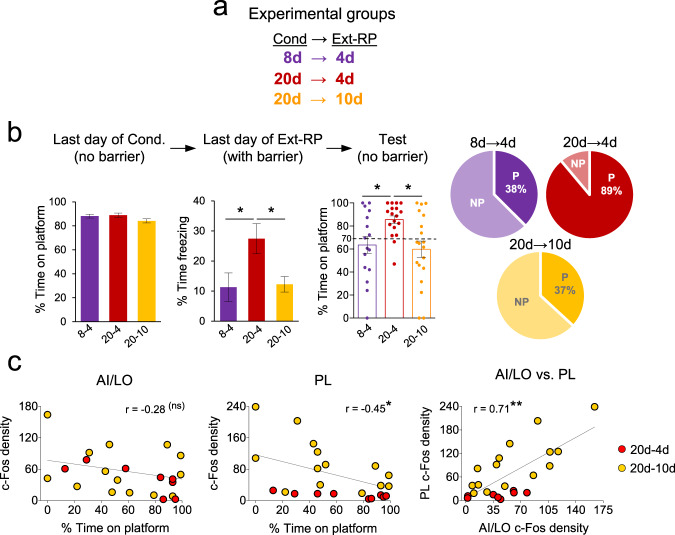
Extending Ext-RP prevented the effects of overtraining. **a** Experimental groups receiving different durations of training for conditioning and Ext-RP phases (8d–4d, 20d–4d, and 20d–10d). **b** While all three groups showed equivalent levels of avoidance at the end of conditioning, 20d–4d showed impaired extinction and persistent avoidance replicating our earlier findings (Fig. 2). However, extending Ext-RP (20d–10d) reduced freezing and avoidance to levels resembling the 8d–4d group. **c** c-Fos analysis revealed a negative correlation between time on platform at Test and AI/LO and PL activity in overtrained rats, as well as a positive correlation between AI/LO and PL activity. This suggests that AI/LO inputs to PL may serve to prevent persistent avoidance following overtraining. Behavior analyses: 8d–4d group: *n* = 16; 20d–4d group: *n* = 18; 20d–10d group: *n* = 19. c-Fos analyses: 20d–4d group: *n* = 8 (AI/LO)−9 (PL); 20–10 group: *n* = 15. Conditioning data shown as average of the last day of conditioning, Ext-RP data shown as a block of the first three trials of the last day of Ext-RP and Test data shown as single trial. All data are shown as mean ± SEM. *p < 0.05, **p < 0.01, ns non-significant.

Because the majority of 20d rats persisted in avoidance following Ext-RP, it could be said that they “failed” Ext-RP. Increased avoidance at test might be expected, however, given the severe impairment in extinction in this group across the 4 days of Ext-RP (see Fig. 2a). We therefore asked if extending Ext-RP beyond 4 days could recover behavior in overtrained rats, by adding a new group in which rats were conditioned for 20d and then given 10d of Ext-RP training. The increase from 4d to 10d was selected to match the increase in conditioning from 8d to 20d (2.5×). To facilitate across-group comparisons, we also trained new sets of animals in the prior two conditions: regular training (8d–4d) and overtraining (20d–4d) (Fig. 5a).

As expected, we replicated our findings of impaired extinction (*F*_(2,50)_ = 4.70, *p* = 0.01) and persistent avoidance (*F*_(2,50)_ = 5.07, *p* < 0.01) in 20d–4d rats compared with 8d–4d rats (all *p*’s < 0.05, one-way ANOVA and Tukey post hoc analyses; Fig. 5b). Importantly, the 20d–10d group resembled the 8d–4d group by exhibiting full extinction by the end of Ext-RP (20d–4d vs. 20d–10d: *p* = 0.03) and low levels of persistent avoidance when the barrier was removed at Test (20d–4d vs. 20d–10d: *p* = 0.01). The percentage of persistent avoidance in the 20d–10d group (37%) matched that of the 8d–4d group (38%), indicating that increasing the duration of Ext-RP eliminated the deleterious effects of overtraining (Fig. 5b). For c-Fos analyses, we focused on AI/LO and PL, two regions previously altered in 8d–4d and 20d–4d groups (see Fig. 4). Combining the two sets of overtrained rats (20d–4d, 20d–10d), we observed that the time spent on the platform (at test) was inversely correlated with activity in PL (*r* = −0.45, *p* = 0.02), and to a lesser extent in AI/LO (*r* = −0.28, *p* = 0.18) suggesting that activity in these areas may act to reduce persistent avoidance in overtrained (but not regularly trained) rats. Furthermore, c-Fos levels in these two areas were positively correlated across rats (*r* = 0.71, *p* < 0.01) (Fig. 5c), consistent with the idea that inputs to PL from AI/LO may serve to reduce persistent avoidance when the barrier is removed, as previously suggested^14^.

## Discussion

In this study, we combined conditioned active avoidance, Ext-RP and c-Fos expression to assess the effects of overtraining on persistent active avoidance. Increasing avoidance training from 8d to 20d did not alter expression of avoidance, but severely impaired the extinction of conditioned freezing during Ext-RP, increased anxiety-like behavior, and triggered long-lasting persistence of avoidance. As for the neuronal activity profile, 8d rats showing persistent avoidance also showed increased activity in regions previously associated with avoidance, as compared with non-persistent rats^17^. Surprisingly, there was no activation of these areas in 20d rats showing persistent avoidance, as compared with the non-persistent rats. Instead, 20d rats showing successful avoidance extinction displayed activation in orbital, insular and thalamic areas. Finally, extending the duration of Ext-RP training prevented the deleterious effects of overtraining. Thus, overtraining re-organizes avoidance circuits and promotes the persistence of avoidance, but this effect can be prevented by extending extinction-based training, and presumably exposure-based therapies for OCD.

### Overtraining impairs extinction of conditioned fear

Despite similar conditioned behavior between groups, 20d rats were impaired in their ability to extinguish the tone-shock association across 4 days of Ext-RP training. Both groups showed equivalently high levels of freezing at the start of Ext-RP, but this extinguished only in the 8d group. Early animal studies reported that increasing avoidance training impaired subsequent extinction, but the duration of training was much less than the 20d used here. Increasing avoidance training from 2 days to 6 days (10 trials per day) delayed extinction of a “jump-up” avoidance task in rats^26^ and dogs^27^, with the latter study also showing elevated anxiety in overtrained animals. These older avoidance tasks are difficult to compare with platform-mediated avoidance (PMA), for which 8 is the minimum number of days required for full conditioning^15^.

Another possible explanation for the extinction deficit could be that blocked access to the platform during Ext-RP had an anxiogenic effect on 20d rats (but not 8d rats) that interfered with extinction of conditioned fear. This is supported by the heightened anxiety exhibited by 20d rats in the EPM following Ext-RP training. While some prior studies suggested that Ext-RP training facilitates subsequent extinction of avoidance^27,28^ others observed a deficit^26^. Prior studies, however, did not examine the effects of extensive overtraining on Ext-RP. Given the larger number of conditioning trials, rats in the 20d group spent more total time on the platform during training than rats in the 8d group (77 min vs. 28 min, *t*_(76)_ = 52.19, *p* < 0.01). This appears to have rendered 20d rats susceptible to enhanced fear and anxiety when the platform was no longer accessible. A final possibility is that overtraining effects may be due to the passage of time between the initiation of conditioning and initiation of Ext-RP (9 days for 8d group vs. 21 days for the 20d group) rather than the additional training trials, as previously observed^29,30^. Our current findings cannot exclude this possibility.

### Overtraining leads to increased avoidance following Ext-RP

As might be expected from the impairment in Pavlovian extinction, 20d rats showed more avoidance than 8d rats when the barrier was removed at test. Indeed, the percentage of rats showing persistent avoidance increased from 37% in the 8d group to 75% in the 20d group, yet there was no group difference in freezing levels. Even prior to tone onset at test, 20d rats expressed heightened avoidance and reduced pressing, suggesting that they may have started the test session in an anxious state. Such a state would favor avoidance, which would serve to reduce passive fear (freezing) to the tone. Thus, overtraining may exacerbate the Ext-RP-induced state of anxiety which then “requires” the expression of avoidance to tamp down fear to the pending stimulus.

In this task, it is difficult to determine whether excessive avoidance in the 20d group was goal-directed (response-outcome) or habitual (stimulus-response). Such a determination would require avoidance to be fully devalued, similar to overfeeding in conditioned foraging tasks^31^ or delivering multiple shocks after removing the avoidance option^32^. Impaired extinction in 20d rats suggests that their avoidance was likely goal-directed, driven by a heightened state of fear and anxiety. Furthermore, the reduction in persistent avoidance after returning the barrier to a corner opposite the platform suggests that 20d rats interpreted the barrier as a negative occasion setter, consistent with goal-directed motivation. If avoidance in 20d rats was goal-directed during Ext-RP, it does not preclude their avoidance from being habitual during conditioning. Indeed, because ERP induces fear/anxiety processes due to the presentation of OCD triggers^33^, the change in sensory cues with Ext-RP may have been sufficient to shift avoidance in 20d rats from habitual back to goal-directed. Nevertheless, extensive avoidance training does not always lead to the development of habits^2^.

### Overtraining alters the avoidance circuit

Prior studies have demonstrated that prefrontal-striatal-amygdala circuitry is activated by, and necessary for, the expression of active avoidance in rodents^17,20,34–36^. However, the neural correlates of persistent avoidance following overtraining had not been studied. We recently reported that expression of PMA is correlated with activation of PL neurons projecting to BLA, whereas extinction of PMA is correlated with activation of IL neurons projecting to BLA and VS^20^. Consistent with this, we have also found that poor extinction of PMA was correlated with excessive activity in PL, BLA, and NAc, and deficient activity in IL^17^. However, when extinction was performed with the platform removed (similar to inserting a barrier), persistent avoidance was not associated with changes in BLA or IL.

The present study replicates this finding using a barrier to block access to the platform: persistent avoiders in the 8d group showed increased activity in PL and NAc, with no changes to BLA or IL (see Table 1). Thus, increased activity in PL and NAc appear to play key roles in the expression of avoidance in rats that are not overtrained^15,17,34,37–39^. Because a naïve control group was not employed in the present c-Fos experiments, we cannot directly compare activity levels to a pre-training baseline. However, comparisons were across subgroups (persistent vs non-persistent), which controls for possible differences in experimental conditions (i.e. handling, context, and shocks received).

A very different picture emerged in overtrained animals. Persistent avoidance in the 20d group was not accompanied by activation of PL, NAc, or amygdala nuclei^30^. Thus, avoidance in the 20d group does not appear to be mediated by the previously described avoidance circuit^35,40^. In fact, persistent 20d rats showed less activity in PL than persistent 8d rats, suggesting that expression of persistent avoidance shifts through different circuits over the course of avoidance training^31,41^. Indeed, a negative correlation between PL activity and avoidance was observed in overtrained rats either receiving 4 or 10 days of Ext-RP. A reduction in cortico-striatal activity might also be interpreted as a reduction in goal-directed control^42,43^. In fact, none of the 20 regions we examined were significantly activated in the 20d rats showing persistent avoidance (see Table 1), however a lack of detectable c-Fos expression difference does not necessarily rule out a role for a given brain region. Nonetheless, our results suggest that overtraining may shift activity away from the prefrontal-striatal circuit controlling adaptive avoidance, perhaps to a less active “default” mode favoring avoidance-like behavior.

Interestingly, the 20d rats which did not avoid at test (non-persistent) showed activation of AI/LO and PVT. The agranular insular-lateral orbital cortex (AI/LO) has been previously implicated in decision making^44,45^, and we previously observed that inactivation of this area induced persistent avoidance^14^. This suggests that activation of AI/LO may be key for maintaining low levels of avoidance following Ext-RP (i.e. extinction transfer). Consistent with this, optogenetic or deep brain stimulation of the AI/LO projections attenuated OCD-like behavior in rodents^46^. The paraventricular nucleus of the thalamus (PVT) has been implicated in both appetitive and aversive behaviors^18,47^ which are in conflict in our task, and PVT is specifically recruited under conditions of approach/avoidance conflict^48^. Because these structures were never activated in 8d rats, this suggests that overtraining may recruit circuits capable of overriding default behaviors under conflict conditions.

### Clinical relevance

In the context of OCD, compulsions often represent anxious avoidance responses. Therefore, each time a compulsion is performed, it might relieve anxiety which would further reinforce avoidance (negative reinforcement)^49–51^. This might resemble the prolonged conditioning in our 20d rats. If anxiety drives compulsions, reducing anxiety with anxiolytic drugs, such as benzodiazepines (BZDs), might be beneficial for OCD treatment^52^. Whereas BZDs have been shown to interfere with context-dependent extinction of conditioned fear^53^, the effectiveness of BZDs in reducing persistent avoidance during exposure-with-response prevention (ERP) in humans remains untested. In humans, extensive overtraining of active avoidance appears to recapitulate core symptoms of OCD such as heightened anxiety, extinction deficits, and persistent avoidance^54^.

Whereas some studies found hyperactivity of the AI/LO hub in OCD^55^, other found hypoactivation. OCD patients displayed reduced serotonin binding in AI/LO^56^ and reduced cognitive flexibility correlated with lower activity in the insular region^7^. Indeed, insular activity is associated with avoiding aversive stimuli and adaptively guiding decision-making processes^49^. Furthermore, damage to the insular cortex is associated with failing to avoid the worst choice^57^. Together, our data suggest that prolonged expression of avoidance may make patients less responsive to exposure-based therapies, consistent with clinical observations^10,12^.

An emerging idea suggests that increasing the length of ERP therapy and/or providing an intensive/residential treatment program represents an effective management approach for severe OCD^58–61^. Thus, patients unresponsive to a brief/standard ERP regimen might benefit from a more intensive course of ERP^62^, resembling the usefulness of extending Ext-RP training in the present study. Similarly, patients applying learned therapeutic strategies in home environments may ameliorate the context-induced relapse of symptoms^63–65^. Extending the duration of ERP therapy may rescue non-responders, possibly by augmenting activity in brain regions that drive non-persistence. An interesting future application of our findings would be to determine if ERP therapy could be facilitated by activating insular, orbital, or thalamic areas with transcranial magnetic or direct current stimulation^66–68^.
